# Supplementary material for: Genomic characterization of Salmonella enterica isolates causing typhoid among Ghanaian patients
Source: PLoS One. 2026 May 12;21(5):e0349142. doi: 10.1371/journal.pone.0349142 (PMC13166912; doi:10.1371/journal.pone.0349142)
Supplement: S1 File — Genome assembly QC metrics; estimation of genome completeness and contamination; percent identity, query converge and ORF conservation of the typhoid-like genes. (PDF) [file pone.0349142.s001.pdf]

S1 Table: Genome Assembly QC metrics by Quast (v 5.3.0)

Isolate	# contigs (>= 25000 bp)	# contigs (>= 50000 bp)	Total length (>= 0 bp)	Total length (>= 50000 bp)	# contigs	Largest contig	Total length	GC (%)	N50	N90	L50	L90	# N's per 100 kbp
UG01	34	26	4705058	4301631	59	466010	4700124	52.1	171996	60678	10	25	0
UG02	35	25	4705701	4186269	62	466010	4699739	52.1	173610	49176	9	26	0
UG08	19	15	4659807	4468581	32	714835	4648656	52.15	393397	104809	5	12	0
UG10	24	19	5050681	4366914	343	773446	4889098	51.89	235526	46235	6	20	0
UG11	24	19	4639519	4367382	44	773446	4622340	52.08	235424	89122	6	17	0
UG12	24	18	4638781	4341124	44	773772	4622115	52.08	235526	91817	6	16	0
UG13	24	19	4640869	4366343	46	773014	4621986	52.08	235850	89122	6	17	0
UG16	19	15	4664562	4461933	31	755300	4648341	52.15	482182	104940	4	12	0
UG17	32	23	4729907	4278673	50	479971	4726125	52.1	204380	56255	9	23	0
UG19	24	18	4639599	4339396	43	773318	4619992	52.08	235526	91817	6	16	0
UG22	15	12	4595236	4443652	29	1151329	4578660	52.18	526842	138502	3	9	0
UG23	14	11	4686021	4543207	32	1308639	4672634	52.27	700859	155402	3	8	0
UG24	34	24	4730581	4221701	55	466575	4724508	52.11	204380	48809	9	25	0
UG25*	7	1	4552914	75722	1269	75722	4552914	51.39	3933	1864	313	996	0
UG26	34	24	4731708	4221159	55	466575	4724794	52.11	204380	48809	9	25	0
UG27	32	24	4699332	4293815	48	457677	4693596	52.09	206043	60678	8	23	0
UG28	11	8	4704848	4573417	20	2003646	4693822	52.24	761554	393480	2	5	0

*Filtered for contigs greater than 1.5Kb

S2 Table: Estimation of genome completeness and contamination using CheckM (v1.2.4).

Bin Id	Marker lineage	# genomes	Completeness	Contamination	Strain heterogeneity
UG01	f__Enterobacteriaceae (UID5103)	157	100	0.08	0
UG02	f__Enterobacteriaceae (UID5103)	157	100	0.08	0
UG08	f__Enterobacteriaceae (UID5103)	157	100	0.08	0
UG10	f__Enterobacteriaceae (UID5103)	157	100	4.53	50
UG11	f__Enterobacteriaceae (UID5103)	157	100	0.44	0
UG12	f__Enterobacteriaceae (UID5103)	157	100	0.44	0
UG13	f__Enterobacteriaceae (UID5103)	157	100	0.44	0
UG16	f__Enterobacteriaceae (UID5103)	157	100	0.08	0
UG17	f__Enterobacteriaceae (UID5103)	157	100	0.08	0
UG19	f__Enterobacteriaceae (UID5103)	157	100	0.44	0
UG22	f__Enterobacteriaceae (UID5103)	157	100	0.08	0
UG23	f__Enterobacteriaceae (UID5103)	157	100	0.08	0
UG24	f__Enterobacteriaceae (UID5103)	157	100	0.08	0
UG26	f__Enterobacteriaceae (UID5103)	157	100	0.08	0
UG27	f__Enterobacteriaceae (UID5103)	157	100	0.08	0
UG28	f__Enterobacteriaceae (UID5103)	157	100	0.08	0
UG25	k__Bacteria (UID203)	5449	100	44.59	89.33
[a]UG25_1kb	f__Enterobacteriaceae (UID203)	119	89.27	9.58	77.88
[b]UG25_1.5kb	f__Enterobacteriaceae (UID203)	119	74.57	4.55	83.64
[c]UG25_2kb	f__Enterobacteriaceae (UID203)	119	63.14	2.57	88.24

[a,b,c] Contigs with less than 1Kb, less than 1.5Kb and less than 2Kb were removed, respectively. The 1.5Kb threshold was selected as the optimal balance to achieve a high-quality contamination profile (<5%) while preserving sufficient genomic completeness for medium-quality draft classification.

S4 Table: Percent identity, query converge and ORF conservation of the typhoid-like genes

Isolate ID	Gene	Identity	Query_Coverage	Expected_Length	Aligned_Length	Intactness	Contig	Start	End
**UG01**	*cdtB*	100.000	100	810	810	Full-Length	NODE_33_length_30970_cov_12.645463	5984	5175
**UG01**	*pltA*	100.000	100	729	729	Full-Length	NODE_33_length_30970_cov_12.645463	3115	3843
**UG01**	*pltB*	100.000	100	414	414	Full-Length	NODE_33_length_30970_cov_12.645463	2685	3098
**UG02**	*cdtB*	100.000	100	810	810	Full-Length	NODE_34_length_30970_cov_9.372694	5984	5175
**UG02**	*pltA*	100.000	100	729	729	Full-Length	NODE_34_length_30970_cov_9.372694	3115	3843
**UG02**	*pltB*	100.000	100	414	414	Full-Length	NODE_34_length_30970_cov_9.372694	2685	3098
**UG08**	*cdtB*	99.877	100	810	810	Full-Length	NODE_8_length_263668_cov_4.282639	5990	5181
**UG08**	*pltA*	98.765	100	729	729	Full-Length	NODE_8_length_263668_cov_4.282639	3120	3848
**UG08**	*pltB*	97.101	100	414	414	Full-Length	NODE_8_length_263668_cov_4.282639	2690	3103
**UG10**	*cdtB*	99.506	100	810	810	Full-Length	NODE_10_length_188284_cov_11.236563	85993	86802
**UG10**	*pltA*	99.451	100	729	729	Full-Length	NODE_10_length_188284_cov_11.236563	88862	88134
**UG10**	*pltB*	98.551	100	414	414	Full-Length	NODE_10_length_188284_cov_11.236563	89292	88879
**UG11**	*cdtB*	99.506	100	810	810	Full-Length	NODE_10_length_188284_cov_9.151145	102292	101483
**UG11**	*pltA*	99.451	100	729	729	Full-Length	NODE_10_length_188284_cov_9.151145	99423	100151
**UG11**	*pltB*	98.551	100	414	414	Full-Length	NODE_10_length_188284_cov_9.151145	98993	99406
**UG12**	*cdtB*	99.506	100	810	810	Full-Length	NODE_10_length_188284_cov_7.094575	102292	101483
**UG12**	*pltA*	99.451	100	729	729	Full-Length	NODE_10_length_188284_cov_7.094575	99423	100151
**UG12**	*pltB*	98.551	100	414	414	Full-Length	NODE_10_length_188284_cov_7.094575	98993	99406
**UG13**	*cdtB*	99.506	100	810	810	Full-Length	NODE_10_length_188284_cov_9.316815	102292	101483
**UG13**	*pltA*	99.451	100	729	729	Full-Length	NODE_10_length_188284_cov_9.316815	99423	100151
**UG13**	*pltB*	98.551	100	414	414	Full-Length	NODE_10_length_188284_cov_9.316815	98993	99406
**UG16**	*cdtB*	99.877	100	810	810	Full-Length	NODE_7_length_263730_cov_5.621435	257737	258546
**UG16**	*pltA*	98.765	100	729	729	Full-Length	NODE_7_length_263730_cov_5.621435	260607	259879
**UG16**	*pltB*	97.101	100	414	414	Full-Length	NODE_7_length_263730_cov_5.621435	261037	260624
**UG17**	*cdtB*	100.000	100	810	810	Full-Length	NODE_31_length_30970_cov_11.845670	5984	5175
**UG17**	*pltA*	100.000	100	729	729	Full-Length	NODE_31_length_30970_cov_11.845670	3115	3843
**UG17**	*pltB*	100.000	100	414	414	Full-Length	NODE_31_length_30970_cov_11.845670	2685	3098
**UG19**	*cdtB*	99.506	100	810	810	Full-Length	NODE_10_length_188284_cov_9.391296	102292	101483
**UG19**	*pltA*	99.451	100	729	729	Full-Length	NODE_10_length_188284_cov_9.391296	99423	100151

**UG19**	*pltB*	98.551	100	414	414	Full-Length	NODE_10_length_188284_cov_9.391296	98993	99406
**UG22**	*cdtB*	97.407	100	810	810	Full-Length	NODE_4_length_485308_cov_7.076916	80828	81637
**UG22**	*pltA*	98.628	100	729	729	Full-Length	NODE_4_length_485308_cov_7.076916	83697	82969
**UG22**	*pltB*	96.860	100	414	414	Full-Length	NODE_4_length_485308_cov_7.076916	84127	83714
**UG24**	*cdtB*	100.000	100	810	810	Full-Length	NODE_33_length_30970_cov_10.911747	5984	5175
**UG24**	*pltA*	100.000	100	729	729	Full-Length	NODE_33_length_30970_cov_10.911747	3115	3843
**UG24**	*pltB*	100.000	100	414	414	Full-Length	NODE_33_length_30970_cov_10.911747	2685	3098
**UG25**	*cdtB*	100.000	100	810	810	Full-Length	NODE_471_length_3145_cov_8.901922	2225	1416
**UG25**	*pltB*	100.000	100	414	414	Full-Length	NODE_378_length_3566_cov_8.066589	2685	3098
**UG26**	*cdtB*	100.000	100	810	810	Full-Length	NODE_33_length_30970_cov_11.131051	5984	5175
**UG26**	*pltA*	100.000	100	729	729	Full-Length	NODE_33_length_30970_cov_11.131051	3115	3843
**UG26**	*pltB*	100.000	100	414	414	Full-Length	NODE_33_length_30970_cov_11.131051	2685	3098
**UG27**	*cdtB*	100.000	100	810	810	Full-Length	NODE_31_length_30969_cov_8.445659	24986	25795
**UG27**	*pltA*	100.000	100	729	729	Full-Length	NODE_31_length_30969_cov_8.445659	27855	27127
**UG27**	*pltB*	100.000	100	414	414	Full-Length	NODE_31_length_30969_cov_8.445659	28285	27872
